# Improving Communication with Patients Discharged from the Emergency Department with Noncardiac Chest Pain: A Scoping Review with Narrative Synthesis

**DOI:** 10.1155/2021/6695210

**Published:** 2021-08-31

**Authors:** Ramzi Shawahna, Aya Ghoul, Najlaa Zaid, Wassan Damrah, Mohammad Jaber

**Affiliations:** ^1^Department of Physiology, Pharmacology and Toxicology, Faculty of Medicine and Health Sciences, An-Najah National University, Nablus, State of Palestine; ^2^An-Najah Biosciences Unit, Centre for Poisons Control, Chemical and Biological Analyses, An-Najah National University, Nablus, State of Palestine; ^3^Department of Medicine, Faculty of Medicine and Health Sciences, An-Najah National University, Nablus, State of Palestine; ^4^An-Najah National Hospital, An-Najah National University, Nablus, State of Palestine

## Abstract

**Background:**

This scoping review with narrative synthesis aimed to analyze scholarly peer-reviewed articles reporting on improving communication with patients discharged from the emergency department with noncardiac chest pain and qualitatively narrate on and summarize items that can be used in guiding communication with patients discharged from the emergency department with noncardiac chest pain.

**Methods:**

The databases of EMBASE/PubMed, Scopus, COCHRANE, CInAHL/EBESCO, UW libraries, and Google Scholar were searched using relevant MeSH and key terms up to February 06, 2020. The selected articles were analyzed for their contents. Items guiding discharge communication were summarized qualitatively.

**Results:**

Twenty-five articles were eligible for full review. These were published in between 1994 and 2020. Of those, 16 (64.0%) originated from the United States and 4 (16%) used some interventional design. A total of 45 different items that could be used in guiding discharge communication with patients presenting to the emergency department with chest pain were identified from the studies included in this review. Items were grouped under 6 categories that were related to initial assessment (8 items), information on diagnosis (7 items), information on discharge (9 items), follow-up suggestions (7 items), symptoms that promote return to the emergency department (7 items), and treatment plan (7 items).

**Conclusion:**

Communication with patients discharged from the emergency department with noncardiac chest pain can be improved. Results of this investigation might be helpful in guiding quality improvement projects aimed for further improvement of communication with patients discharged from the emergency department with noncardiac chest pain.

## 1. Introduction

Despite the advancements and innovations in diagnostic and interventional methods, evaluation of patients who present to the emergency department with chest pain is still challenging [1–3]. Patients who present to the emergency department with chest pain are often thought to have acute coronary syndrome which is a frequent serious health concern among emergency patients [4, 5]. Of the patients who present to the emergency department with chest pain, about 20% will receive a diagnosis of acute coronary syndrome and require prolonged stay at the hospital. However, the underlying cause of chest pain in the majority (about 80%) of the patients will be attributed to a noncardiac condition that is usually not threatening to the life of the patients [1, 4]. After evaluation and risk stratification, patients whose chest pain was attributed to a noncardiac cause can be discharged from the emergency department either to home or to another outpatient management setting [6, 7].

Improving quality of care, patient experiences, and patient satisfaction are high priorities in all healthcare settings [8, 9]. Improving communication between patients and their healthcare providers is crucial in improving the quality of care [10–12]. Recently, improving communication in emergency department has attracted considerable attention [11, 12]. Following evaluation and at discharge, healthcare providers should communicate information to patients and/or their attendants on the diagnosis made, treatment options, recommendations for follow-up, self-care, and red flags prompting return to the emergency department. In emergency departments, the environment could be chaotic in which healthcare providers often deal with a large number of mentally and/or physically frail patients. As a result, communication between patients and their healthcare providers in many cases could be poor and ineffective. Ineffective communication could jeopardize the quality of healthcare delivery and patient satisfaction [13, 14]. On the other hand, effective communication was shown to contribute to empowering patients to understand and recall information and recommendations provided by their healthcare providers. Additionally, effective communication can have positive impact on anxiety [15–17], adherence to treatments/recommendations [16, 18], treatment outcomes [16, 19, 20], satisfaction [20], and reassurance [12].

Despite the importance of communication between patients and their healthcare providers in the emergency department articulated in previous research, little scoping and narrative synthesis of scholarly peer-reviewed articles reporting on improving communication with patients who present to the emergency department with noncardiac chest pain was conducted before.

Scoping reviews with narrative analysis have evolved as useful tools in analyzing the contents of scholarly peer-reviewed literature on a particular subject [21]. In this study, we aimed to conduct a comprehensive scoping review of scholarly peer-reviewed articles reporting on improving communication with patients who present to the emergency department with noncardiac chest pain. Additionally, this review aimed to identify, collect, analyze, qualitatively narrate on, and summarize items that can be used in guiding communication with patients discharged from the emergency department with noncardiac chest pain.

## 2. Methods

### 2.1. Study Design

This scoping review with narrative synthesis is being reported in adherence to the Preferred Reporting Items for Systematic Reviews and Meta-Analyses Statement for Scoping Reviews (PRISMA-ScR) [22]. Adherence to the PRISMA-ScR checklist is shown in Supplementary Materials (Supplementary Table S1). Previous scoping reviews informed the development of the protocol used in scoping part of this study [21, 23].

### 2.2. Search for Articles

A systematic search for articles was conducted to identify and select studies reporting on improving discharge communication with patients who present to the emergency department with chest pain. The following databases were searched: Excerpta Medica database (EMBASE) through PubMed, Scopus, COCHRANE, Cumulative Index to Nursing and Allied Health Literature (CInAHL) hosted by EBESCO, and UW libraries. The databases were searched using medical subject headings (MeSH) and key terms relevant to improving discharge communication with patients who present to the emergency department with chest pain: “patient discharge,” “patient discharge summaries,” “communication,” “teach-back communication,” “health communication,” “hospital communication systems,” “communication barriers,” “physician-patient relations,” “therapeutic alliance,” “patient satisfaction,” “emergency service, hospital,” “emergency medical services,” “emergency treatment,” and “chest pain.” MeSH and key terms were combined using the Boolean operators “AND” and “OR” [21, 24–29]. The search approach was customized for each database used in this study. To identify more studies, we also manually searched the references of the studies identified through the databases. To supplement the search, Google Scholar was used as a search engine to search and identify potentially relevant articles that were not indexed in the databases used. The databases were searched as late as February 06, 2020.

A manual search was performed using the titles and abstracts of the articles identified through the search to decide on which studies will be selected for full-text review.

### 2.3. Selection of Articles

Three researchers (AG, NZ, and WD) independently performed the literature search. The literature search was supervised by RS (PhD) who had prior knowledge and experience in searching the databases used in this study [21]. Results of the literature search were imported into EndNote X7 (Clarivate Analytics, Philadelphia) in the form of Research Information Systems (RIS) files. Duplicate studies were removed. The imported studies were screened against the inclusion and exclusion criteria by three researchers (AG, NZ, and WD) independently. To ensure reproducibility of the results, each researcher repeated the process three times. Discussions and consensus were initiated to resolve discrepancies. All authors (AG, NZ, WD, and RS) agreed on the final studies that would be included in the bibliometric analysis and qualitative synthesis.

#### 2.3.1. Inclusion Criteria

In this study, articles were included when they reported original studies on communication with patients who present to the emergency department with chest pain. The search was not restricted to any particular country, year of publication, and/or publication status. Articles were included regardless of the methods used. Articles with mention of discharge communication, emergency department, and chest pain were given a priority for inclusion in the full-text review.

#### 2.3.2. Exclusion Criteria

Articles published in languages other than English were not included. Editorials, commentaries, letters to the editor, and review articles were excluded. Studies that were not related to communication in emergency department in relation to chest pain were also excluded.

### 2.4. Content Analysis and Extraction of Items

In this study, a form was created in Excel spreadsheet (Microsoft Inc.) to collect the data. Three researchers (AG, NZ, and WD) independently reviewed the full text of the selected articles. Items relevant to communication with patients complaining of chest pain within the full text of each article were highlighted using Adobe Acrobat Pro (Adobe Inc., California) by each researcher independently. The researchers extracted items independently into the data collection form. Items were then shared between all researchers, and results were compared. Conflicting results and discrepancies were resolved by discussion and consensus. The extracted items were analyzed and organized thematically [21, 30].

In this study, data relevant to name of author (s), year of publication, country/setting in which the study was conducted, aims of the study, design of the study, study participants, method data collection, main findings, and funding were collected. The data collection form is found in Supplementary Materials (Supplementary Table S2).

Due to the nature and heterogeneity of the results, a narrative synthesis was used to present the results of the scoping part of this study. Items that can be used in guiding discharge communication with patients presenting to the emergency department with chest pain were qualitatively synthesized. From the synthesized items, the authors selected the most important key messages that could be used in guiding discharge communication with patients presenting to the emergency department with chest pain. Discussions, deliberations, and consensus were used to select the most important key messages.

## 3. Results

### 3.1. Results of the Literature Search

**Figure 1:**
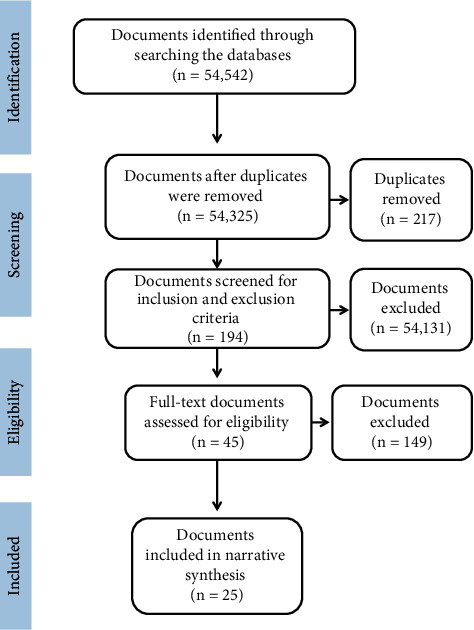
PRISMA flow diagram of study selection.

The literature search in the databases yielded a total of 54,542 documents. When the duplicates were removed, 54,325 documents were retained. Upon applying the inclusion and exclusion criteria, 194 documents were retained. Of those, 45 were eligible for full-text review. Of those, 25 articles were included in the narrative synthesis. Of the selected articles, 10 (40.0%) were open access and 15 (60.0%) were accessible by subscription. Details of the search strategy are shown in Figure 1.

### 3.2. Characteristics of the Selected Articles

#### 3.2.1. Year of Publication

**Figure 2:**
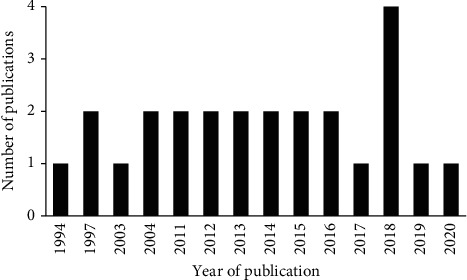
Year and number of publications for the selected studies (*n* = 25).

The selected studies were published in the years 1994 to 2020. Of all studies, 4 (16.0%) were published in the year 2018. Details of the year of publication are shown in Figure 2.

#### 3.2.2. Location/Country in Which the Study Was Conducted

**Figure 3:**
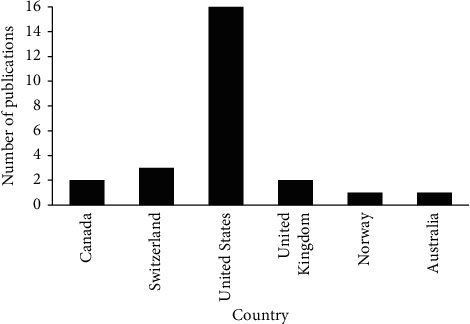
Countries in which the selected studies were conducted (*n* = 25).

Of the selected studies, 16 (64.0%) were conducted in the United States and 3 (12.0%) were conducted in Switzerland. Details of the countries in which the selected studies were conducted are shown in Figure 3.

#### 3.2.3. Study Design and Tools

Of the studies selected, 4 (16%) used some interventional design. The rest of the studies were observational or qualitative. Details of the study design and tools used in the selected articles are shown in Supplementary Materials (Supplementary Table S3). Narrative summaries of the selected articles are shown in Supplementary Materials (Supplementary Table S4).

#### 3.2.4. Source of Funding

**Figure 4:**
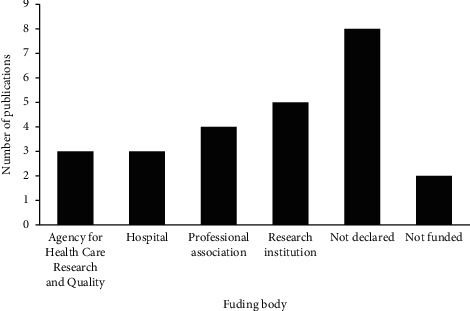
Sources of funding of the selected studies (*n* = 25).

Of the studies selected for this scoping review, 5 (20.0%) were funded by a research institution, 4 (16.0%) were funded by a professional association, and 3 (12.0%) were funded by Agency for Health Care Research and Quality. Details of the funding bodies are shown in Figure 4.

### 3.3. Summary of Items Guiding Discharge Communication with Patients Presenting to the Emergency Department with Chest Pain

Table 1 lists 45 different items that could be used in guiding discharge communication with patients presenting to the emergency department with chest pain that were identified from the studies included in this review. Items were grouped under 6 categories that were related to initial assessment (8 items), information on diagnosis (7 items), information on discharge (9 items), follow-up suggestions (7 items), symptoms that promote return to the emergency department (7 items), and treatment plan (7 items). Details of these items are shown in Table 1.

**Figure 5:**
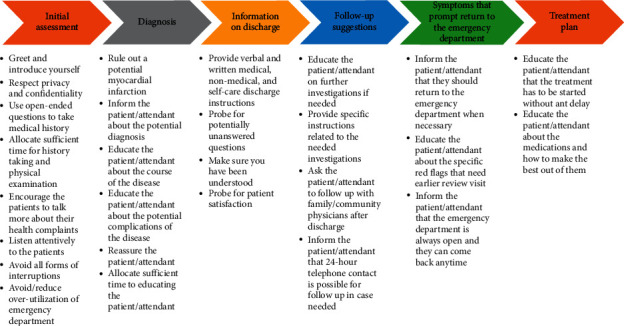
The most important key messages that can be used in guiding discharge communication.

The most important key messages that could be used in guiding discharge communication with patients presenting to the emergency department with chest pain that were selected by the authors are shown in Figure 5.

### 3.4. Summary of Methods Used to Assess Satisfaction of Patients with the Discharge Communication

**Figure 6:**
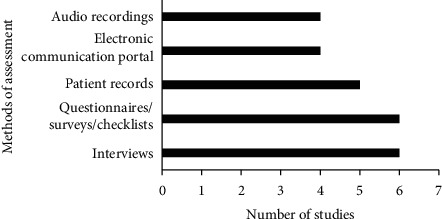
Methods used to assess satisfaction of patients with the discharge communication.

The studies included reported different methods that can be used to assess the level of patient satisfaction with discharge communication. These methods included interviews with the patients, using questionnaires/surveys/checklists, reviewing patient records, using some sort of electronic communication portal, and listening to audio recordings of the emergency department.  Figure 6 shows the number of times these methods were reported in the selected studies.

## 4. Discussion

In modern healthcare systems, communication between healthcare providers and patients has received considerable attention. Additionally, there has been more emphasis on improving satisfaction of patients being discharged after receiving necessary healthcare services. The purpose of this scoping with narrative synthesis was to identify, analyze, and summarize peer-reviewed articles published on improving discharge communication with patients who presented to the emergency department with chest pain. Findings of this study portrayed the scholarly literature on improving discharge communication with patients who presented to the emergency department with chest pain and summarized items that could be used in guiding discharge communication with patients presenting to the emergency department with chest pain. To our knowledge, this study is the first appraisal of peer-reviewed scholarly articles reporting on improving discharge communication with patients who presented to the emergency department with chest pain. Additionally, this is the first study to summarize items that could be used in guiding discharge communication with patients presenting to the emergency department with chest pain.

In this study, original articles were selected and included. In scholarly peer-reviewed publications, the majority of the articles are published as original articles [31, 32]. Of the articles selected in this study, more than half (60.0%) were accessible by subscription and the rest were open access. Despite the fact that open access publications are increasingly becoming popular in scholarly peer-reviewed literature, the majority of the peer-reviewed articles are still accessible by subscription [33].

The articles selected in this study were retrieved through a thorough search of 5 main large databases of scholarly published peer-review literature. The search engine Google Scholar was also used to supplement the search. The databases used in this study are known for the quality of the journals indexed in each database. Additionally, these databases are commonly used in scoping and systematic reviews [21, 34].

The articles selected in this study were published over the year span of 1994–2020, and the majority of the articles (76.0%) were published beyond the year 2010. Growth of the number of articles in recent years could have indicated more emphasis on improving communication with and satisfaction of patients with chest pain being discharged from emergency departments.

In the present study, the majority (64.0%) of the articles reported studies conducted in the Unites States. Articles also reported studies conducted in the United Kingdom, Canada, Australia, Switzerland, and Norway. In this study, none of the studies included originated from a developing country. Our findings could have been explained by the high productivity of developed countries compared to productivity of developing countries in terms of research and scholarly peer-reviewed literature [35]. It has been argued that productivity of research and scholarly peer-reviewed literature can be affected by many factors including infrastructure, funding, equipment, and availability of skilled researchers [36].

Of the studies selected, 15 (60.0%) declared receiving funds from research institutions, professional associations, or agencies for healthcare research and quality. Funding could be crucial in supporting and sustaining scientific/academic research and productivity of scholarly peer-reviewed literature productivity [37–40].

Items guiding discharge communication with patients presenting to the emergency department with chest pain were summarized and grouped under 6 categories: initial assessment, information on diagnosis, information on discharge, follow-up suggestions, symptoms that prompt return to the emergency department, and information on treatment. In emergency department, assessment of patients complaining of chest pain should be initiated in a timely manner in quiet places to preserve the privacy and confidentiality of the patient [41]. Recent studies have shown that timely access to specialist cardiology assessment improved quality of healthcare services, experiences, and satisfaction of patients with chest pain [8]. In general, patients were discharged with high level of satisfaction with the quality of care provided, comfort, communication, engagement, and minimal uncertainty in the diagnosis. This could be achieved by using open-ended questions with appropriate prompts to take the history and spending sufficient time in performing investigations during which the patients should be given the time to talk about their complaints. Healthcare providers should provide the patients/attendants with complete information on the examinations performed and the diagnoses made. This could contribute to improving patient experiences and satisfaction [8]. The patients/attendants should be informed with the potential cause of their chest pain and the course of the disease with the potential complications. Before discharge, patients/attendants should be reassured that the investigations allowed the healthcare providers to rule out a myocardial infarction before the patient can be discharged. In emergency department, the main interventions used to reduce suffering of patients are (a) reassurance, (b) diagnosis, (c) explanation, and (d) advice [42]. In their recent study, Ferry et al. proposed a model in which communication interventions include providing patients with information relevant to investigations, actively listening to their complaints, and acknowledging their health concerns [12]. Using such models could promote trust between the healthcare provider and the patient and might be helpful in reassuring patients. Whenever the patient is ready for discharge, the patients/attendants should be notified by the healthcare providers. Discharge information along with written and verbal instructions including medical, nonmedical, and self-care instructions should be provided to the patients/attendants. Patients/attendants should be given the opportunity to ask questions and healthcare providers should ensure that the patients/attendants understood and are satisfied with the discharge information provided and can recall them. In general, healthcare providers often overestimate the ability of the patients to recall instructions [18, 19]. Patients/attendants should be informed whether more investigations were needed, why and when these investigations were needed, and where and how these investigations can be done. Patients/attendants should be advised to consult/follow up with their family/community physicians, when, and how to follow up. Patients/attendants should be informed of the signs, symptoms, and red flags that would prompt a return to the emergency department. Patients/attendants should be informed what, when, and how to take treatment and how to make the best out of the treatment.

Patient satisfaction with discharge communication can be measured using different methods. Healthcare providers/stakeholders can conduct face-to-face or phone interviews with the patients [12, 18, 19, 43–45]. Satisfaction of the patients can be gauged using appropriately designed open- or close-ended questions. Moreover, healthcare providers/stakeholders can use prevalidated self- or interviewer-administered questionnaires/surveys/checklists to measure the level of patient satisfaction with the provided discharge communication [11, 18, 19, 44, 46–52]. Auditors/healthcare providers could also review records of the patients to assess the quality of the communication and predict satisfaction of the patients [53–57]. Additionally, auditors/healthcare could listen to audio recordings of the emergency department and assess the quality of the communication [15, 16, 53]. Healthcare establishments could also use some sort of electronic communication portal to allow patients to report their satisfaction/dissatisfaction with the discharge communication [52, 58, 59].

### 4.1. Strengths and Limitations of the Study

This scoping with narrative synthesis provided adequate coverage of scholarly peer-reviewed research on improving discharge communication with patients who presented to the emergency department with chest pain and summarized items that could be used in guiding discharge communication with patients presenting to the emergency department with chest pain. The main databases indexing the largest number of peer-reviewed literature were used in the search [60]. Findings of this study could be useful for decision and policy makers interested in improving discharge communication and satisfaction of patients with chest pain. This study was the first to combine scoping, content analysis, and narrative synthesis methods to address the width and depth of studies that were published as peer-reviewed scholarly research articles on improving discharge communication with patients presenting to the emergency department with chest pain. The study summarized items guiding discharge communication with patients presenting to the emergency department with chest pain and highlighted the hot research topics in the field. Findings of this study could be useful in shaping and directing future research aiming to improve discharge communication with patients presenting to the emergency department with chest pain.

Findings of this study could be interpreted taking into consideration the following limitations. First, articles published in languages other than English were excluded from this study. As we restricted the search to articles published in English, we could have missed some interesting findings in articles published in languages other than English. Second, a scoping method was used for the literature search and review. Compared to other review approaches including the scoping approach, the systematic approach has been advertised as the most robust in preserving rigor and reproducible results. However, in this study we did not opt for a systematic review approach because the nature, aims, questions, problem, intervention, comparison, outcome, study design (PICOS), and number of articles needed for this study encouraged a scoping approach [61–63]. Third, we did not assess the scientific quality of the studies included in this investigation using appropriate tools. Assessing the quality of the studies included could have been interesting in adding another dimension to the findings of the present study. However, quality assessments are often performed in systematic reviews rather than scoping reviews.

## 5. Conclusion

In summary, effective communication and patient satisfaction are major concerns in the emergency department, especially those presenting with chest pain. Poor communication between patients and healthcare providers could have devastating consequences on the quality of healthcare services provided and health outcomes of the patients. This scoping study provided insights into the width and depth of scholarly peer-reviewed documents on improving discharge communication with patients presenting to the emergency department with chest pain. Results of this investigation might be helpful in directing future research for further improvement of discharge communication with patients presenting to the emergency department with chest pain. More studies are still needed to address poor communication and improve satisfaction of patients presenting to the emergency department with chest pain.

## Figures and Tables

**Table 1:** Items guiding discharge communication with patients presenting to the emergency department with chest pain.

#	Items
*Initial assessment*	
1	Assessments should take place in quiet and private places/sides. Privacy and confidentiality of the patients should be respected.
2	Healthcare providers should greet and introduce themselves to patients and/or their accompanying attendants. In all cases, patients and their accompanying attendants should be treated with dignity and respect.
3	Medical history should be taken using open-ended questions with appropriate prompts whenever needed to clarify/probe for something.
4	Healthcare providers should spend sufficient time during history taking and physical examination.
5	Patients should be encouraged to talk more about their health complaints.
6	Healthcare providers should listen attentively while patients explain their complaints.
7	All forms of interruptions should be avoided or at least minimized.
8	Efforts should be made to avoid overutilization of emergency department.

*Information on diagnosis*	
1	Healthcare providers should explain to the patients and/or their accompanying attendants that the cardiovascular, pulmonary, and blood circulation systems were carefully examined.
2	Healthcare providers should inform the patients and/or their accompanying attendants that all the investigations had ruled out myocardial infarction at this time.
3	Healthcare providers should inform the patients and/or their accompanying attendants about the potential diagnosis.
4	Healthcare providers should explain to the patients and/or their accompanying attendants the course of the disease.
5	Healthcare providers should explain to the patients and/or their accompanying attendants the potential complications of the disease.
6	Healthcare providers should reassure the patients and/or their accompanying attendants.
7	Healthcare providers should spend sufficient time providing information to the patients and/or their accompanying attendants.

*Information on discharge*	
1	Healthcare providers should notify the patients that they are ready to be discharged home.
2	Healthcare providers should give discharge instructions to the patients and/or their accompanying attendants.
3	Healthcare providers should give both written and verbal instructions to the patients and/or their accompanying attendants.
4	Healthcare providers should provide nonmedical instructions like avoiding stress, taking rest, etc.
5	Healthcare providers should provide self-care instructions like quitting smoking, eating healthy diet, adherence to regular exercise, control of other comorbidities like hypertension and diabetes, etc., if present.
6	Healthcare providers should ask the patients and/or their accompanying attendants if they have more unanswered questions.
7	Healthcare providers should make sure that the patients and/or their accompanying attendants understood the information provided.
8	Healthcare providers should make sure that the patients and/or their accompanying attendants are satisfied with the discharge information.
9	Healthcare providers should determine if the diagnosis and discharge information can be correctly recalled by the patients and/or their accompanying attendants immediately after discharge.

*Follow-up suggestions*	
1	Healthcare providers should inform the patients and/or their accompanying attendants what further investigations are still or will be needed.
2	Healthcare providers should explain to the patients and/or their accompanying attendants the reasons why further investigations are still or will be needed.
3	Healthcare providers should inform the patients and/or their accompanying attendants when and where the investigations can be done.
4	Healthcare providers should explain specific instructions related to the needed investigations like if the patient should come fasting, fed, etc.
5	Healthcare providers should advise the patients to consult/follow up with their family/community physicians after discharge.
6	Healthcare providers should inform the patients when and how to follow up.
7	Healthcare providers should inform the patients that 24-hour telephone contact is possible for follow-up in case needed.

*Symptoms that prompt return to the emergency department*	
1	Healthcare providers should inform the patients that returning to the emergency department is an option when necessary.
2	Healthcare providers should inform the patients that they should return to the emergency department if their chest pain lasted for more than 10 minutes.
3	Healthcare providers should explain to the patients specific red flags that need earlier review visit like fever, focal neurological deficit, sweating, etc.
4	Healthcare providers should inform the patients to return to the emergency department in case of chest pain that is radiated to jaw or arms.
5	Healthcare providers should inform the patients to return to the emergency department if they have difficulty breathing.
6	Healthcare providers should inform the patients to return immediately to the emergency department if they started to complain of chest pain that did not respond to nitroglycerin.
7	Healthcare providers should inform the patients that the emergency department is always open and they can come back anytime, even at night, during weekends, and holidays.

*Treatment plan*	
1	Healthcare providers should inform the patients that the treatment has to start without any delay.
2	Healthcare providers should tell the patients the name of prescribed medication (acetyl-salicylic acid, beta-blockers, nitroglycerin, etc.).
3	Healthcare providers should tell the patients the dose of the prescribed medication that they should take.
4	Healthcare providers should tell the patients the frequency of the prescribed medication at which they should take.
5	Healthcare providers should tell the patients when to take the prescribed medication in relation to meals.
6	Healthcare providers should tell the patients the potential adverse reactions that could be associated with the prescribed medication and how to cope with them.
7	Healthcare providers should tell the patients what to avoid when taking the prescribed medications and how to make the best out of them.

## Data Availability

All data relevant to this work are included within the manuscript or available as supplementary materials.

## Abbreviations

PRISMA-ScR: Systematic Reviews and Meta-Analyses Statement for Scoping Reviews
EMBASE: Excerpta Medica database
CInAHL: Cumulative Index to Nursing and Allied Health Literature
MeSH: Medical subject headings
PICOS: Problem, intervention, comparison, outcome, study design
RIS: Research Information Systems.

## Supplementary Materials

Supplementary Materials Supplementary Table S1: adherence to the Preferred Reporting Items for Systematic Reviews and Meta-Analyses Statement for Scoping Reviews (PRISMA-ScR). Supplementary Table S2: the data collection form. Supplementary Table S3: study design and tools used in the selected articles. Supplementary Table S4: narrative summaries of the articles selected in this study (*n* = 25).
