# Supplementary material for: Improving Communication with Patients Discharged from the Emergency Department with Noncardiac Chest Pain: A Scoping Review with Narrative Synthesis
Source: Emerg Med Int. 2021 Aug 31;2021:6695210. doi: 10.1155/2021/6695210 (PMC8426084; doi:10.1155/2021/6695210)
Supplement: Supplementary Materials — Supplementary Table S1: adherence to the Preferred Reporting Items for Systematic Reviews and Meta-Analyses Statement for Scoping Reviews (PRISMA-ScR). Supplementary Table S2: the data collection form. Supplementary Table S3: study design and tools used in the selected articles. Supplementary Table S4: narrative summaries of the articles selected in this study (n = 25). [file 6695210.f1.docx]

**Supplementary Materials**

**Supplementary Table S1:** Adherence to the Preferred Reporting Items for Systematic Reviews and Meta-Analyses statement for scoping reviews (PRISMA-ScR)([Tricco *et al.* 2018](#_ENREF_21))

| **Section** | **Item #** | **PRISMA-ScR checklist item** | **Reported on page #** |
| --- | --- | --- | --- |
| **Title** |  |  |  |
| **Title** | 1 | Identify the report as a scoping analysis. | The scoping part of this work is referred to in the title |
| **Abstract** |  |  |  |
| **Structured summary** | 2 | Provide a structured summary including, as applicable: background, objectives, eligibility criteria, sources of evidence, charting methods, results and conclusions that relate to the review question(s) and objective(s). | Provided and structured in accordance with the journal's style. Please see the abstract for background, objectives, inclusion criteria, data extraction, results, and conclusion. |
| **Introduction** |  |  |  |
| **Rationale** | 3 | Describe the rationale for the review in the context of what is already known. Explain why the review question(s)/objective(s) lend themselves to a scoping review approach. | Provided. Please see the Introduction (Importance) section, Page 2 Lines 13-23. |
| **Objectives** | 4 | Provide an explicit statement of the question(s) and objective(s) being addressed with reference to their key elements (e.g., population or participants, concepts and context), or other relevant key elements used to conceptualize the review question(s) and/or objective(s)). | Provided. Please see the Introduction (Goals of This Investigation), Page 2 Lines 33-40. |
| **Methods** |  |  |  |
| **Protocol and registration** | 5 | Indicate if a review protocol exists, if and where it can be accessed (e.g., web address), and, if available, provide registration information including registration number. | Protocol was not registered. Description of the protocol followed is provided with references. Please see Methods section, Page 3, Lines 2-7. |
| **Eligibility criteria** | 6 | Specify the characteristics of the sources of evidence (e.g., years considered, language, publication status) used as criteria for eligibility, and provide a rationale. | Provided. Please see the Methods section, Page 3, Lines 8-26. |
| **Information sources** | 7 | Describe all information sources (e.g., databases with dates of coverage, contact with authors to identify additional sources) in the search, as well as the date the most recent search was executed. | Provided. Please see the Methods section, Page 3, Lines 9-24. |
| **Search** | 8 | Present the full electronic search strategy for at least one database, including any limits used, such that it could be repeated. | Provided. Please see the Methods section, Page 3, Lines 9-24. |
| **Selection of sources of evidence** | 9 | State the process for selecting sources of evidence (i.e., screening, eligibility) included in the scoping review. | Provided. Please see the Methods section, Page 3, Lines 27-36. |
| **Data charting process** | 10 | Describe the methods of charting data from the included sources of evidence (e.g., piloted forms; forms that have been tested by the team before their use, whether data charting was done independently, in duplicate) and any processes for obtaining and confirming data from investigators. | Provided. Please see the Methods section, Page 3, Lines 27-36, Page 4 Lines 21-32. |
| **Data items** | 11 | List and define all variables for which data were sought and any assumptions and simplifications made. | Provided. Please see the Methods section, Page 3, Lines 27-36. |
| **Critical appraisal of individual sources of evidence** | 12 | ***If done,*** provide a rationale for conducting a critical appraisal of included sources of evidence; describe the methods used and how this information was used in any data synthesis (if appropriate). | Provided. Please see the Methods section, Pages 3 and 4 and supplementary data (appendix E7). |
| **Summary measures** | 13 | *Not applicable for scoping reviews.* |  |
| **Synthesis of results** | 14 | Describe the methods of handling and summarizing the data that were charted. | Provided. Please see the Methods section, Page 3, Lines 27-36 and Page 4 Lines 8-32. |
| **Risk of bias across studies** | 15 | *Not applicable for scoping reviews.* |  |
| **Additional analyses** | 16 | *Not applicable for scoping reviews.* |  |
| **Results** |  |  |  |
| **Selection of sources of evidence** | 17 | Give numbers of sources of evidence screened, assessed for eligibility, and included in the review, with reasons for exclusions at each stage, ideally using a flow diagram. | Provided. Please see the Results section, Page 5, Lines 3-7 and Supplementary Data Appendix E3 for the PRISMA flow chart. |
| **Characteristics of sources of evidence** | 18 | For each source of evidence, present characteristics for which data were charted and provide the citations. | Provided. Please see the Results section, Page 5-6, Tables 1-4 and Supplementary data. |
| **Critical appraisal within sources of evidence** | 19 | ***If done***, present data on critical appraisal of included sources of evidence (see item 12). | Please see Tables 1-4 and Supplementary data. |
| **Results of individual sources of evidence** | 20 | For each included source of evidence, present the relevant data that were charted that relate to the review question(s) and objective(s). | Please see Tables 1-4 and supplementary data (appendix E7). |
| **Synthesis of results** | 21 | Summarize and/or present the charting results as they relate to the review question(s) and objective(s). | Provided. Please see the Results section, Pages 5-6 and supplementary data. |
| Risk of bias across studies | 22 | *Not applicable for scoping reviews.* |  |
| Additional analyses | 23 | *Not applicable for scoping reviews.* |  |
| **Discussion** |  |  |  |
| Summary of evidence | 24 | Summarize the main results (including an overview of concepts, themes, and types of evidence available), explain how they relate to the review question(s) and objectives, and consider the relevance to key groups. | Provided. Please see the Discussion section, Page 7, Lines 2-16. |
| Limitations | 25 | Discuss the limitations of the scoping review process. | Provided. Please see the Discussion section, Page 9, Lines 19-29. |
| Conclusions | 26 | Provide a general interpretation of the results with respect to the review question(s) and objective(s), as well as potential implications and/or next steps. | Provided. Please see the Discussion section, Page 9, Lines 30-38. |
| **Funding** |  |  |  |
| Funding | 27 | Describe sources of funding for the included sources of evidence, as well as sources of funding for the scoping review. Describe the role of the funders of the scoping review. | Provided at the end of the manuscript |

**Supplementary Table S2:** The data collection form

| **#** | **Author(s)** | **Publication year** | **Setting and/or country** | **Objectives of the study** | **Study design** | **Participants** | **Data collection** | **Analysis** | **Main findings** | **Funding** |
| --- | --- | --- | --- | --- | --- | --- | --- | --- | --- | --- |
| 1 |  |  |  |  |  |  |  |  |  |  |
| 2 |  |  |  |  |  |  |  |  |  |  |
| 3 |  |  |  |  |  |  |  |  |  |  |
| 4 |  |  |  |  |  |  |  |  |  |  |
| 5 |  |  |  |  |  |  |  |  |  |  |
| 6 |  |  |  |  |  |  |  |  |  |  |
| 7 |  |  |  |  |  |  |  |  |  |  |
| 8 |  |  |  |  |  |  |  |  |  |  |
| 9 |  |  |  |  |  |  |  |  |  |  |
| 10 |  |  |  |  |  |  |  |  |  |  |
| 11 |  |  |  |  |  |  |  |  |  |  |
| 12 |  |  |  |  |  |  |  |  |  |  |
| 13 |  |  |  |  |  |  |  |  |  |  |
| 14 |  |  |  |  |  |  |  |  |  |  |
| 15 |  |  |  |  |  |  |  |  |  |  |
| 16 |  |  |  |  |  |  |  |  |  |  |
| 17 |  |  |  |  |  |  |  |  |  |  |
| 18 |  |  |  |  |  |  |  |  |  |  |
| 19 |  |  |  |  |  |  |  |  |  |  |
| 20 |  |  |  |  |  |  |  |  |  |  |
| 21 |  |  |  |  |  |  |  |  |  |  |
| 22 |  |  |  |  |  |  |  |  |  |  |
| 23 |  |  |  |  |  |  |  |  |  |  |
| 24 |  |  |  |  |  |  |  |  |  |  |
| 25 |  |  |  |  |  |  |  |  |  |  |

**Supplementary Table S3:** Study design and tools used in the selected articles

| **#** | **Author(s)** | **Year** | **Study design** | **Study tool** |
| --- | --- | --- | --- | --- |
| **Studies related to development and/or assessment of quality and content of communication** | | | | |
| 1 | Marty et al([Marty *et al.* 2013](#_ENREF_13)) | 2013 | Observational, cross-sectional | Interviews, questionnaire |
| 2 | Vashi and Rhodes([Vashi & Rhodes 2011](#_ENREF_22)) | 2011 | Observational, retrospective, descriptive | Audio recordings of emergency departments |
| 3 | Rhodes et al([Rhodes *et al.* 2004](#_ENREF_17)) | 2004 | Observational, retrospective, descriptive | Audio recordings of patient encounters at an emergency department |
| 4 | Crane([Crane 1997](#_ENREF_6)) | 1997 | Observational, cross-sectional | Questionnaire, interviews |
| 5 | Ackermann et al([Ackermann *et al.* 2016](#_ENREF_2)) | 2016 | Observational, cross-sectional, qualitative | Face-to-face interviews, lists, and questionnaires |
| 6 | Ackermann et al([Ackermann *et al.* 2012](#_ENREF_1)) | 2012 | Observational, cross-sectional | A case vignette |
| 7 | Burman et al([Burman *et al.* 2011](#_ENREF_4)) | 2011 | Observational, prospective over a period of 3 months | Records and data from emergency medical communication centers, ambulances and primary care doctors |
| 8 | Musey et al([Musey *et al.* 2018](#_ENREF_14)) | 2018 | Observational, cross-sectional | A questionnaire |
| 9 | Newman et al([Newman *et al.* 2015](#_ENREF_15)) | 2015 | Observational, cross-sectional | Matched-pair surveys |
| 10 | Kuhlman et al([Kuhlman *et al.* 2019](#_ENREF_12)) | 2019 | Mixed method | Review of literature and data, a written consensus algorithm, pre- and post-analysis |
| 11 | Wen et al([Wen *et al.* 2015](#_ENREF_25)) | 2013 | Observational, retrospective | Patient chart review |
| 12 | Christenson et al([Christenson *et al.* 2004](#_ENREF_5)) | 2004 | Observational, prospective | Patient records and testing results |
| **Studies related to satisfaction with communication** | | | | |
| 1 | Rydman et al([Rydman *et al.* 1997](#_ENREF_18)) | 1997 | Interventional, randomized | A diagnostic protocol |
| 2 | Probst et al([Probst *et al.* 2018](#_ENREF_16)) | 2018 | Interventional, secondary analysis of a clinical trial | A scale to measure involvement of patients in decision making and another scale to measure desire of patients for involvement in decision making. |
| 3 | White([White 2003](#_ENREF_26)) | 2003 | Observational, qualitative | A modified grounded theory approach, interviews |
| **Studies related to development, implementation, and/or evaluation of a system or a protocol** | | | | |
| 1 | Hunchak et al([Hunchak *et al.* 2015](#_ENREF_10)) | 2015 | Observational, descriptive | A web-based communication portal |
| 2 | Jacobs et al([Jacobs *et al.* 2012](#_ENREF_11)) | 2012 | Observational, cross-sectional | Electronic medical and billing records |
| 3 | Alley et al([Alley *et al.* 2018](#_ENREF_3)) | 2018 | Interventional, pre- and post-tests | A multi-disciplinary, problem-based learning workshop |
| 4 | Scott et al([Scott *et al.* 2014](#_ENREF_19)) | 2014 | Observational, comparative | An evidence-based Chest Pain Management Service and clinical pathway |
| 5 | Scott et al([Scott *et al.* 2017](#_ENREF_20)) | 2017 | Observational, retrospective descriptive | The US International Classification of Diseases, 9th Revision, Clinical Modification [ICD-9-CM]) to the Medical Priority Dispatch System triage codes |
| 6 | Watkins and Patrician([Watkins & Patrician 2014](#_ENREF_23)) | 2014 | Observational, retrospective comparative | Electronic Emergency Provider Written Plan of Discharge template |
| 7 | Ferry et al([Ferry *et al.* 2020](#_ENREF_7)) | 2020 | Observational, qualitative | Interviews |
| 8 | Helmchen et al([Helmchen *et al.* 2016](#_ENREF_9)) | 2016 | Observational, comparative | Patient records submitted to a central statewide repository |
| 9 | Gesell et al([Gesell *et al.* 2018](#_ENREF_8)) | 2018 | Observational, qualitative | Interviews |
| 10 | Weingarten et al([Weingarten *et al.* 1994](#_ENREF_24)) | 1994 | Interventional, prospective, controlled clinical trial | Personalized written and verbal guideline, recommendation, and reminders |

**Supplementary Table S4:** Narrative summaries of the articles selected in this study (*n = 25*)

| **#** | **Author(s)** | **Year** | **Study objective(s)** | **Focus group/participants/sample size** | **Data collected** | **Main findings** | **Funding source** |  | **Study site/Country** |  |  |  |
| --- | --- | --- | --- | --- | --- | --- | --- | --- | --- | --- | --- | --- |
| **Studies related to development and/or assessment of quality and content of communication** | | | | | | | | | | | |  |
| 1 | Marty et al([Marty *et al.* 2013](#_ENREF_13)) | 2013 | To investigate: (1) how healthcare providers educated patients comprehensively upon discharge, 2) how exactly patients recalled information relevant to their health conditions after discharge, 3) how informed the patients were when they were discharged from the emergency department, and 4) if patient satisfaction was related to informedness. | Patients discharged from emergency department (*n = 93*) | Patients were interviewed for 5-10 min after discharge by the researchers. Patients answered questions and provided information relevant to their diagnosis, tests scheduled, and follow-ups. Both the physician and the patient scored their satisfaction with the transmission of information on a 5-point scale during the discharge dialogue (1: very dissatisfied; 5: very satisfied). | Adequate discharge communication was prevalent in 83% of the cases. Patients correctly remembered 82% of the diagnostic details, 56% of the scheduled tests, and 72% of the follow-ups. Medication details were most vulnerable to be forgotten or for distortion. Overall, 43% of patients left the emergency department fully informed about the diagnosis, scheduled exams, and follow-ups. Ratings for patient satisfaction were high and did not correlate with informedness. | Not declared |  | Emergency department of a university hospital, Switzerland |  |  |  |
| 2 | Vashi and Rhodes([Vashi & Rhodes 2011](#_ENREF_22)) | 2011 | To assess the quality and contents of verbal discharge information provided at two emergency departments using quantitative and qualitative methods. | The study was a secondary analysis of emergency department recordings (*n = 844*) of communication with patients. Of those, 477 contained discharge instructions. | The audiotapes were coded for the following data relevant to discharged communication: 1) diagnosis, 2) prognosis, 3) self-care, 4) pharmacotherapy, 5) symptoms prompting a return to the emergency department, (6) dates for follow-ups, (7) follow-ups on pharmacotherapy, (8) opportunities to ask questions, and (9) confirmation of patient comprehension. | Only 22% of providers of discharge information have verified if the patients understood the discharge information. Verbal discharge communication at the emergency department were often insufficient. Limited opportunities were offered to most patients to ask questions and/or clarify understandings. | Agency for Healthcare Research and Quality |  | Emergency departments (n = 2) at an urban academic tertiary medical center and a suburban community hospital, United States |  |  |  |
| 3 | Rhodes et al([Rhodes *et al.* 2004](#_ENREF_17)) | 2004 | To assess communication at an emergency department relevant to timing, quality of taking the medical history, physical examination, and providing discharge instructions | Audio recordings of patient encounters (*n = 93*) at an emergency department which included: 62 cases of taking medical history and physical examinations and 31 discharge instructions | Audiotapes were analyzed and the data collected included: 1) the average time of taking patient history and physical examination, 2) external interruptions occurred during taking history or physical examination, 3) if the history taking and physical examination occurred at the same time, and 4) whether the clinician provided patients with information relevant to future expectations, education, or advice relevant to behavior and risk factors. | On average, history taking and physical examination took about 7.5 min (range 1 to 20 min). Resident physicians introduced themselves in about 75% of encounters with patients presenting to the emergency department. Resident physicians disclosed their training status in 8% of the times. Few patients (20%) explained their presenting complaints with no interruptions. Discharge instructions were received in 76 seconds (range 7-202 seconds). Discussions on diagnosis, course of illness, self-care, treatment, time for follow-up, and red flag to return to the emergency department were each discussed in less than 65% of the time. A minority (16%) of the patients were given the opportunity to ask questions. | Agency for Health Care Research and Quality |  | An emergency department at an inner-city academic medical center, United States |  |  |  |
| 4 | Crane([Crane 1997](#_ENREF_6)) | 1997 | To evaluate patients who were discharged from emergency department whether they understood their diagnosis, recommended medications, additional guidance, and follow-up with treatment plans | Patients discharged from emergency department (*n = 314*) | Over a period of two weeks, a convenience sample was taken from patients who were discharged from the emergency department. One of four bilingual research assistants interviewed the patients using a questionnaire. | The patients discharged from the emergency department who were interviewed could correctly identify about 59% of the instructions given to them. Most of patients (63.8%) regarded the clinicians as their primary source of information. Spanish speaking patients could identify less instructions compared to English speaking patients. | Not declared |  | Kern Medical Center, California, United States |  |  |  |
| 5 | Ackermann et al([Ackermann *et al.* 2016](#_ENREF_2)) | 2016 | To define the ideal contents of discharge communication with patients who present to the emergency department with chest pain | Clinicians (*n = 47*); patients (*n = 51*) | Views of physicians and patients on the ideal contents of discharge communication at the emergency department were exposed qualitatively. Consensus-based items guiding discharge communication with patients who present to the emergency department with chest pain. | A list containing 34 items was developed. The items were grouped into 5 categories: 1) communication relevant to diagnosis, 2) follow-up instructions, 3) self-care instructions, 4) warnings and red flags, and 5) treatment plan. | University Hospital, Basel, Switzerland |  | University Hospital of Basel, Switzerland |  |  |  |
| 6 | Ackermann et al([Ackermann et al. 2012](#_ENREF_1)) | 2012 | To identify items that physicians thought were crucial in communicating discharge information to patients in a time restrained (15 min) interaction. | Internists (*n = 38*) and emergency physicians (*n = 9*) | The study participants were presented with a case vignette of a patients with chest pain and a list containing 81 items that could be communicated to the patient. The study physicians were asked to prioritize and pick the most important ones to convey in a 15 min interaction. The time required to convey each item was estimated by experts (*n = 7*). | Physicians picked on average 36 items from the list (range: 20–57). Experts estimated that 44.5 min were needed to communicate the items. The number of items picked by emergency physicians was not significantly different from the number of items that were picked by internists (31.6 ± 6.2 vs. 37.4 ± 10.2). Experts estimated that the time required to share the selected items by the emergency physicians was significantly shorter compared to the time required to share the items picked by the internists (36.9 ± 6.3 vs. 46.4 ± 13.5). The selected items were relevant to: 1) suspected diagnosis, 2) addressing risk factors, 3) the need for further examination and alternative investigations, and 4) consequences in case of positive tests. | Scientific Fund of the Emergency Department, University Hospital Basel, Switzerland |  | University Hospital of Basel, Switzerland |  |  |  |
| 7 | Burman et al([Burman et al. 2011](#_ENREF_4)) | 2011 | To obtain and analyze data relevant to the epidemiology of acute chest pain outside Norwegian hospitals through the data of emergency medical communication centers. | Patients in red response situations (*n = 5,180*, of whom 1104 had chest pain) | Data were collected prospectively from 3 emergency medical communication centers covering 816,000 citizens over three months. Emergency medical communication centers collected information relevant to situations that were triaged as red response “acute response with highest priority”. More data were collected from ambulances and primary care physicians. In the analysis, codes of the International Classification of Primary Care-2 symptoms and scores of the National Committee on Aeronautics were applied. | Findings of the study showed that the annual incidence of chest pain was 5.4 per 1,000 citizens. Almost 1 in 4 patients had a condition that was life threatening. The median prehospital response time was 13 min. Most of the patients (76%) were admitted to hospitals for further assessment. About 10% of the patients received no further investigations. | National Centre for Emergency Primary Health Care, Uni Health, Bergen, the Norwegian Medical Association’s fund for Research in General Practice |  | Haugesund, Stavanger and Innlandet hospitals, Norway |  |  |  |
| 8 | Musey et al([Musey et al. 2018](#_ENREF_14)) | 2018 | To explore opinions, beliefs, and practices of healthcare providers in emergency departments with regard to patients complaining of low-risk chest pain as believed secondary to stress and anxiety. | Healthcare providers (*n = 409*) who were residents, nurses, and physician assistant in from 46 states and 7 countries | A modified Delphi technique was used to develop a list of 22 items. | Healthcare providers believed that about 30% of the patients presenting to the emergency department with chest pain were at low risk for acute coronary syndrome. Less than half of the healthcare providers addressed stress and anxiety as an explanation of chest pain. The study highlighted opportunities to improve communication in emergency departments. | Not funded |  | American College of Emergency Physicians Scientific Assembly in Las Vegas, United States |  |  |  |
| 9 | Newman et al([Newman et al. 2015](#_ENREF_15)) | 2015 | To assess physician-patient communication on the risks of potential acute coronary syndrome and to evaluate the contents of discussions that surround disposition decision. | Patient-physician survey pairs (n = 425 patients) | The sample was obtained from 2 academic inner-city emergency departments. After organization conversation, research assistants conducted matched-pair surveys of patients with possible acute coronary syndrome and their physicians and communicated risk approximates and aim of admission. | The majority of the patients (65%) reported discussing with the physician the likelihood of myocardial infarction as a cause of their symptoms. Less than half (45%) of the physician reported discussing with the patient the likelihood of myocardial infarction as a cause of their symptoms. The study reported poor communication, patients and physicians over-rate both the risk myocardial infarction and possible benefit of hospital admission. Communication that surrounds disposition decisions in patients with chest pain could be ineffective or misleading. | Not declared |  | Academic inner-city emergency departments, United States |  |  |  |
| 10 | Kuhlman et al([Kuhlman et al. 2019](#_ENREF_12)) | 2019 | To use a mixed method to review evidence, share practice experience, and formulate a written algorithm with key decision points to establish the standard-of-care of patients presenting to the emergency department with chest pain. | Patients with chest pain (n = 200,691) | The mixed method was used to establish the standard-of-care for patients presenting to the emergency department with chest pain. The literature and data were assessed and reviewed. Consensus algorithm was developed to detect compliance. A pre- and post-analyses were performed in a 9-campus hospital system. | Implementation of the standard of care increased discharge of patients from the emergency department by 99%, decreased inpatient admissions by 63%, and decreased those going into observation status by 20%. | Not funded |  | AdventHealth Orlando, Florida, United States |  |  |  |
| 11 | Wen et al([Wen et al. 2015](#_ENREF_25)) | 2013 | To determine the percentage of patients admitted to the emergency department who were discharged with a symptomatic or pathological diagnosis | Patients visiting the emergency department (n = 797) with chest pain, abdominal pain or headache | Two reviewers reviewed charts of patients visiting the emergency department with chest pain, abdominal pain or headache. Based on the discharge diagnosis, the reviewers coded each chart as either symptomatic or pathological diagnosis. | Of the patients with chest pain, 17% (95% CI 12% to 22%) received pathological discharge diagnosis. Findings of the study suggested that the majority of the patients presenting to the emergency department were discharged without a pathological diagnosis that could explain their symptoms. | Massachusetts American College of Emergency Physicians and Department of Emergency Medicine at Brigham & Women’s Hospital |  | Massachusetts General Hospital, United States |  |  |  |
| 12 | Christenson et al([Christenson et al. 2004](#_ENREF_5)) | 2004 | To identify the percentages of patients who presented to emergency department with chest pain who were discharged inappropriately despite having an acute coronary syndrome or those who did not have an acute coronary syndrome and were held for further testing and investigations. | Patients with chest pain (n = 1819) | Research nurses collected information relevant to pain onset time, time of admission and discharge, vital signs, risk factors, disposition, period of hospital admission, findings of the cardiac consultations, diagnosis, medications, electrocardiogram results, results of laboratory investigations, and adverse events. | Of the patients with chest pain, 13.2% received a 30-day diagnosis of acute myocardial infarction and 8.6% received a diagnosis of unstable angina. Of those, 5.3% were discharged from the emergency department without diagnosis with acute coronary syndrome or further investigations required. Detecting acute coronary syndrome was clinically sensitive in 94.7% of the cases and was specific in 73.8% of the cases. | Canadian Institutes of Health Research |  | St. Paul's and Vancouver General hospitals, Canada |  |  |  |
| **Studies related to satisfaction with communication** | | | | | | | | | | | | |
| 1 | Rydman et al([Rydman et al. 1997](#_ENREF_18)) | 1997 | To compare satisfaction with a diagnostic protocol of patients who presented to the emergency department with chest pain compared to standard inpatient hospitalization | Patients with chest pain (n = 104), randomized into chest pain observation unit protocol (n = 52) or hospital inpatient (control) (n = 52) | Randomized patients were interviewed by research interviewers at the time of discharge from the hospital for inpatients (control) or at the end of the diagnostic procedure for the patients in the chest pain observation unit protocol. | Patients who were diagnosed using the chest pain observation unit protocol were more satisfied than patients who were in the control group (inpatient hospitalization). The study suggested improving standard practice by taking into consideration clinical and cost outcomes between two care alternative methods of management. | Agency for Health Care Policy and Research |  | Illinois, United States |  |  |  |
| 2 | Probst et al([Probst et al. 2018](#_ENREF_16)) | 2018 | To examine the factors that were correlated with actual and desired patient involvement in clinical decisions among patients presenting to the emergency department with chest pain. | Patients presented to the emergency department and physicians (n=898) | Two scales were used in the study. A scale was used to measure involvement of patients in decision making and another scale was used to measure desire of patients for involvement in decision making. | Involvement of patients in decision making scores were predicted by the study site and using a decision aid. Scores were higher for patients who were randomized to the decision aid group when compared with those who were randomized to the usual care group. | Investigator-initiated contract from the Patient-Centered Outcomes Research Institute |  | Emergency departments at: 1) University of California Davis, 2) Mayo Clinic Rochester, 3) Indiana University, 4) University of Pennsylvania, 5) Thomas Jefferson University, and 6) Mayo Clinic Jacksonville, United States |  |  |  |
| 3 | White([White 2003](#_ENREF_26)) | 2003 | To assess initial reactions of patients admitted to the emergency department with chest pain. | Patients admitted to the hospital with chest pain (n = 25) and followed through discharge (n = 10) | Interviews were conducted with the patients through fieldwork. Data collection and analysis were directed using an interpretive grounded theory. | The themes emerged explained three types of interactions: 1) supportive, 2) controlling, and 3) informative. The study suggested that discussions could be a key factor in shaping experiences of patients admitted to the hospital with chest pain. | Not declared |  | A small district general hospital in the North of England, United Kingdom |  |  |  |
| **Studies related to development, implementation, and/or evaluation of a system or a protocol** | | | | | | | | | | | | |
| 1 | Hunchak et al([Hunchak et al. 2015](#_ENREF_10)) | 2015 | To design and pilot test an online alert system that electronically notify family physicians of their patients visits to emergency departments and provided access to laboratory tests and information relevant to diagnosis | Family physicians (n = 9) for whom records of 270 patients were reviewed with 880 patient visits to the emergency department | The pilot study was conducted in three phases: 1) pre-pilot chart audit to estimate how often family physicians received information on their patients' visits to the emergency departments at baseline, 2) the family physicians using web portal were monitored online, and 3) data on family physicians who used the web portal were collected quantitatively (written scanning) and qualitatively (focus group). | The use of the web portal increased receipt of information relevant to patient emergency department visits by 17%. All family physicians were ‘‘often’’ or ‘‘always’’ aware of their patients' visits to the emergency departments. An enhancement in the emergency department -family physician coordination was shown after the development and introduction of the web-based emergency department visit communication tool. The effect of this system on enhancement of quality of treatment, timeliness of follow-up, and decreased duplication of investigations. | Not declared |  | Mount Sinai Hospital, Toronto, Canada |  |  |  |
| 2 | Jacobs et al([Jacobs et al. 2012](#_ENREF_11)) | 2012 | To assess the effect of a change in policy from using telephonic and face-to-face interpreting to using video-interpreting networks on care provision in emergency department. | Hospital A: English speaker before using video network (n=2850), Post video network (n=3957), Hospital A: Spanish speaker, previous video network (n=1727), Post video network (n=2454); Hospital B: English speaker before using network (n=2767), Post using network (n=2781), Spanish speaker, before using network (n=734), Post using network (n=681), Patient being admitted primarily for acute coronary syndrome (n=425), physician (n=415) | Data were extracted from electronic medical and billing records to compare between English- and Spanish-speaking patients presenting with chest pain and abdominal pain before and after using of video-interpreting networks with regard to time and admission rates in emergency department. | The study showed that mean time and rate of admission to emergency department at both language groups and both hospitals decreased in in the post-video interpreting network period compared with the previous one, and using video interpreting network had minimal effect on health care in emergency department. | California HealthCare Foundation |  | Hospitals in California, United States |  |  |  |
| 3 | Alley et al([Alley *et al.* 2018](#_ENREF_3)) | 2018 | To study the improvement on medical students' knowledge after multi-disciplinary, problem-based learning workshop about cardiac risk stratification and assessment of risks for patients presenting to emergency departments with chest pain. | 3rd year medical students (n=219) | Pre- and post-retention tests were used to evaluate the acquisition and retention of information by the medical students who participated in a chest pain risk stratification multi-disciplinary, problem-based learning workshop. | 3rd year medical students participated in an educational experience to acquire experience and understanding of how to care in a systems-based manner for patients presenting to the emergency department with chest pain. After the workshop, students obtained a 19.7% increase in post-test scores (95% confidence interval [17.3-22.2%]) compared to pre-testing. Additionally, students maintained an improvement of 11.1% (95% confidence interval [7.2-15.0%]) on a retention test compared to the pre-test. | Donaghue Foundation with support from the Association of American Medical Colleges, Duke Endowment, Abbott Point of Care, and Roche Diagnostics for investigator-initiated research |  | Wake Forest School of Medicine, Winston-Salem, United States |  |  |  |
| 4 | Scott et al([Scott et al. 2014](#_ENREF_19)) | 2014 | To identify the efficiency of an evidence-based chest pain management protocol and clinical pathway in delivering timely and suitable triage as well as safe and effective patient care that decreased unnecessary overnight admission and the load on hospital resources. | Patient with chest pain (n = 5662) | An evidence-based Chest Pain Management Service and clinical pathway were developed and implemented, including the introduction of after-hours exercise stress test. | The Induction of the chest pain management protocol and the exercise stress testing service led to earlier discharge for low-risk patients (5181 admission nights) and avoided 1360 admission days for high-risk patients as a result of receiving timely diagnosis and treatment. The protocol also allowed appropriate treatment of patients with positive and equivocal exercise stress test results. | Not declared |  | Royal Brisbane and Women’s Hospital, Australia |  |  |  |
| 5 | Scott et al([Scott et al. 2017](#_ENREF_20)) | 2017 | To compare hospital-confirmed definite diagnoses to all calls handled by emergency medical dispatchers using the Chest Pain/Chest Discomfort (Non-Traumatic) Chief Complaint Protocol. | Data from Utah Department of Health (n = 65,975); Data from medical dispatch (n = 106,595) | The retrospective descriptive study used emergency medical dispatch, emergency medical service, and hospital datasets at two emergency centers. Patients who presented by chest pain according to protocol and to hospital datasets were included. The cases grouped as ischemic heart disease, other cardiac-related condition or non-cardiac-related conditions associated with chest pain. | Patients with chest pain (3,007 cases) were identified. Chest pain caused by a wide range of etiologies. Age, gender, previous history of ischemic heart disease, severe breathing problems and clamminess are all potential factors in identifying a possible or likely ischemic heart disease event. Respiratory causes were the most common cause of the non-cardiac chest pain. Cardiac conditions were the highest triage level the majority of the time. | Not declared |  | Emergency communication centers (n = 2) in Salt Lake County and Salt Lake City Fire Department, United States |  |  |  |
| 6 | Watkins and Patrician([Watkins & Patrician 2014](#_ENREF_23)) | 2014 | To evaluate implementation of an electronic Emergency Provider Written Plan of Discharge form that was used to notify primary care providers to follow-up with patients discharged from emergency departments with low-risk chest pain to primary care as the result of lack and/or omissions in follow-up care for patients. | Patients with chest pain (n = 358) ; Control group (n = 132); Intervention (n = 226) | Computerized Patient Record System database was used to extract and evaluate data on patients with chest pain. | The study showed that the majority (93.8%) of the patients in the intervention group received additional testing and 92.5% of them were followed up by primary healthcare providers. Follow-up for this population increased significantly after using the electronic Emergency Provider Written Plan of Discharge form the study provided evidence that standardizing handoff communication from the emergency to primary care providers can get better the quality of patient care by ensuring timely diagnostic and follow-up care. | Not declared |  | Jackson Department of Veteran Affairs Medical Center, United States |  |  |  |
| 7 | Ferry et al([Ferry et al. 2020](#_ENREF_7)) | 2020 | To explore the effectiveness of implementing an early rule-out step might affect experience of patients presenting with chest pain | Patients with chest pain before (n = 23) or after (n = 26) introducing of an early rule-out step | Patients who presented to the emergency department with chest pain who were suspected of having an acute coronary syndrome for whom myocardial infarction was ruled out were interviewed before and after implementing an early rule-out step. | Thematic analysis led to a number of themes that included: 1) seeking healthcare advice before visiting the emergency department, 2) discrepancies in troponin results being interpreted by the clinicians and experiences of patients with regard to their illness, 3) trusting the healthcare providers, 4) active listening might enhance reassurance in case of negative results, 5) routine care procedures as a source of frustration. | Edinburgh and Lothians Health Foundation, British Heart Foundation |  | United Kingdom |  |  |  |
| 8 | Helmchen et al([Helmchen et al. 2016](#_ENREF_9)) | 2016 | To assess of the communication‐and‐resolution program that was introduced to address unexpected adverse outcomes in caring for patients could justify costs and trajectories of use | Records of patients diagnosed with chest pain (n = 140,347) | Propensity-score matched discharges from hospitals that were used as intervention and comparative. Changes in legal environment, technology, price, and protocol of treatment were accounted for. | There was an increase in patients diagnosed with chest pain in the intervention hospital compared to the comparison hospitals or the pre-implementation period. Growth rates and costs of clinical laboratory testing and radiology investigations in the intervention hospital were reduced by 3.8% and 6.9% points. | Agency for Healthcare Research and Quality |  | Hospitals in Illinois, United States |  |  |  |
| 9 | Gesell et al([Gesell et al. 2018](#_ENREF_8)) | 2018 | To explore barriers and facilitators of implementing the HEART Pathway which is an evidence-based decision tool that could be used to identify patients presenting to the emergency department with chest pain who could be discharged early with the objective of reducing unhelpful or harmful hospitalization. | Semi-structured interviews were conducted with stakeholders (n = 25) who were health system leaders or emergency department physicians | The Consolidated Framework for Implementation Research framework-driven deductive approach was used to code and analyze the interviews. | The study showed that time, limited resources, challenges of the electronic health record system, engagement of the stakeholders, and concerns of the patients were potential barriers to implement the HEART Pathway. However, reducing length of stay, unhelpful testing, avoiding iatrogenic complications, user-friendliness, and support of evidence-based decisions were potential facilitators to implement the HEART Pathway. | National Center for Advancing Translational Sciences, National Institutes of Health |  | Academic medical centers in North and South Carolina, United States |  |  |  |
| 10 | Weingarten et al([Weingarten et al. 1994](#_ENREF_24)) | 1994 | To evaluate the use of practice guidelines and reminders on reducing duration of hospital stay for patients with chest pain | Patients with chest pain (n = 375) who presented to coronary care and intermediate care units | Healthcare providers (clinicians) who provided care for patients with chest pain who were considered at low risk for complications received personalized, written, and verbal reminders with regard to a guideline recommending a 2-day hospital stay. | Using the guideline recommendations and reminders increased adherence by 50%-69% and resulted in a 0.91-day (95% CI, 0.18 to 1.63) reduction in the length of hospital stay for all patients with chest pain who were at low risk for complications. Direct and indirect costs were reduced by $1397/per patient (CI, $176 to $2618). | American Heart Association |  | Cedars-Sinai Medical Center, West Los Angeles; United States |  |  |  |

**References**

Ackermann S, Bingisser MB, Heierle A, Langewitz W, Hertwig R & Bingisser R (2012): Discharge communication in the emergency department: physicians underestimate the time needed. *Swiss Med Wkly* **142**, w13588.

Ackermann S, Heierle A, Bingisser MB, Hertwig R, Padiyath R, Nickel CH, Langewitz W & Bingisser R (2016): Discharge Communication in Patients Presenting to the Emergency Department With Chest Pain: Defining the Ideal Content. *Health Commun* **31**, 557-565.

Alley WD, Burns C, Hartman ND, Askew K & Mahler SA (2018): 3 for the Price of 1: Teaching Chest Pain Risk Stratification in a Multidisciplinary, Problem-based Learning Workshop. *The western journal of emergency medicine* **19**, 613-618.

Burman RA, Zakariassen E & Hunskaar S (2011): Acute chest pain - a prospective population based study of contacts to Norwegian emergency medical communication centres. *BMC Emerg Med* **11**, 9.

Christenson J, Innes G, McKnight D, Boychuk B, Grafstein E, Thompson CR, Rosenberg F, Anis AH, Gin K, Tilley J, Wong H & Singer J (2004): Safety and efficiency of emergency department assessment of chest discomfort. *CMAJ : Canadian Medical Association journal = journal de l'Association medicale canadienne* **170**, 1803-1807.

Crane JA (1997): Patient comprehension of doctor-patient communication on discharge from the emergency department. *J Emerg Med* **15**, 1-7.

Ferry AV, Strachan FE, Stewart SD, Marshall L, Lee KK, Anand A, Shah ASV, Chapman AR, Mills NL & Cunningham-Burley S (2020): Exploring Patient Experience of Chest Pain Before and After Implementation of an Early Rule-Out Pathway for Myocardial Infarction: A Qualitative Study. *Ann Emerg Med* **75**, 502-513.

Gesell SB, Golden SL, Limkakeng AT, Jr., Carr CM, Matuskowitz A, Smith LM & Mahler SA (2018): Implementation of the HEART Pathway: Using the Consolidated Framework for Implementation Research. *Crit Pathw Cardiol* **17**, 191-200.

Helmchen LA, Lambert BL & McDonald TB (2016): Changes in Physician Practice Patterns after Implementation of a Communication-and-Resolution Program. *Health Serv Res* **51 Suppl 3**, 2516-2536.

Hunchak C, Tannenbaum D, Roberts M, Shah T, Tisma P, Ovens H & Borgundvaag B (2015): Closing the circle of care: implementation of a web-based communication tool to improve emergency department discharge communication with family physicians. *Canadian Journal of Emergency Medicine* **17**, 123-130.

Jacobs EA, Fu PC, Jr. & Rathouz PJ (2012): Does a video-interpreting network improve delivery of care in the emergency department? *Health Serv Res* **47**, 509-522.

Kuhlman J, Moorhead D, Kerpchar J, Peach DJ, Ahmad S & O'Brien PB (2019): Clinical Transformation Through Change Management Case Study: Chest Pain in the Emergency Department. *EClinicalMedicine* **10**, 78-83.

Marty H, Bogenstätter Y, Franc G, Tschan F & Zimmermann H (2013): How well informed are patients when leaving the emergency department? comparing information provided and information retained. *Emerg Med J* **30**, 53-57.

Musey PI, Jr., Lee JA, Hall CA & Kline JA (2018): Anxiety about anxiety: a survey of emergency department provider beliefs and practices regarding anxiety-associated low risk chest pain. *BMC Emerg Med* **18**, 10.

Newman DH, Ackerman B, Kraushar ML, Lederhandler MH, Masri A, Starikov A, Tsao DT, Meyers HP & Shah KH (2015): Quantifying Patient-Physician Communication and Perceptions of Risk During Admissions for Possible Acute Coronary Syndromes. *Ann Emerg Med* **66**, 13-18, 18.e11.

Probst MA, Tschatscher CF, Lohse CM, Fernanda Bellolio M & Hess EP (2018): Factors Associated With Patient Involvement in Emergency Care Decisions: A Secondary Analysis of the Chest Pain Choice Multicenter Randomized Trial. *Acad Emerg Med* **25**, 1107-1117.

Rhodes KV, Vieth T, He T, Miller A, Howes DS, Bailey O, Walter J, Frankel R & Levinson W (2004): Resuscitating the physician-patient relationship: emergency department communication in an academic medical center. *Ann Emerg Med* **44**, 262-267.

Rydman RJ, Zalenski RJ, Roberts RR, Albrecht GA, Misiewicz VM, Kampe LM & McCarren M (1997): Patient satisfaction with an emergency department chest pain observation unit. *Ann Emerg Med* **29**, 109-115.

Scott AC, O'Dwyer KM, Cullen L, Brown A, Denaro C & Parsonage W (2014): Implementation of a chest pain management service improves patient care and reduces length of stay. *Crit Pathw Cardiol* **13**, 9-13.

Scott G, Clawson JJ, Gardett I, Broadbent M, Williams N, Fivaz C, Marshall G, Barron T & Olola C (2017): 9-1-1 Triage of Non-Traumatic Chest Pain: Association with Hospital Diagnosis. *Prehosp Emerg Care* **21**, 525-534.

Tricco AC, Lillie E, Zarin W, O'Brien KK, Colquhoun H, Levac D, Moher D, Peters MDJ, Horsley T, Weeks L, Hempel S, Akl EA, Chang C, McGowan J, Stewart L, Hartling L, Aldcroft A, Wilson MG, Garritty C, Lewin S, Godfrey CM, Macdonald MT, Langlois EV, Soares-Weiser K, Moriarty J, Clifford T, Tuncalp O & Straus SE (2018): PRISMA Extension for Scoping Reviews (PRISMA-ScR): Checklist and Explanation. *Ann Intern Med* **169**, 467-473.

Vashi A & Rhodes KV (2011): "Sign right here and you're good to go": a content analysis of audiotaped emergency department discharge instructions. *Ann Emerg Med* **57**, 315-322.e311.

Watkins LM & Patrician PA (2014): Handoff communication from the emergency department to primary care. *Adv Emerg Nurs J* **36**, 44-51.

Weingarten SR, Riedinger MS, Conner L, Lee TH, Hoffman I, Johnson B & Ellrodt AG (1994): Practice guidelines and reminders to reduce duration of hospital stay for patients with chest pain. An interventional trial. *Ann Intern Med* **120**, 257-263.

Wen LS, Espinola JA, Kosowsky JM & Camargo CA, Jr. (2015): Do emergency department patients receive a pathological diagnosis? A nationally-representative sample. *The western journal of emergency medicine* **16**, 50-54.

White AK (2003): Interactions between nurses and men admitted with chest pain. *Eur J Cardiovasc Nurs* **2**, 47-55.
